# 24-Month Outcomes of Primary Care Web-Based Depression Prevention Intervention in Adolescents: Randomized Clinical Trial

**DOI:** 10.2196/16802

**Published:** 2020-10-28

**Authors:** Benjamin Van Voorhees, Tracy R G Gladstone, Kunmi Sobowale, C Hendricks Brown, David A Aaby, Daniela A Terrizzi, Jason Canel, Eumene Ching, Anita D Berry, James Cantorna, Milton Eder, William Beardslee, Marian Fitzgibbon, Monika Marko-Holguin, Linda Schiffer, Miae Lee, Sarah A de Forest, Emily E Sykes, Jennifer H Suor, Theodore J Crawford, Katie L Burkhouse, Brady C Goodwin, Carl Bell

**Affiliations:** 1 Department of General Pediatrics University of Illinois at Chicago College of Medicine Chicago, IL United States; 2 The Robert S and Grace W Stone Primary Prevention Initiatives Wellesley Centers for Women Wellesley College Boston, MA United States; 3 Department of Psychiatry Yale School of Medicine New Haven, CT United States; 4 Department of Psychiatry and Behavioral Sciences Feinberg School of Medicine Northwestern University Chicago, IL United States; 5 Department of Preventive Medicine Feinberg School of Medicine Northwestern University Chicago, IL United States; 6 NorthShore University Health System Evanston, IL United States; 7 Harvard Vanguard Cambridge, MA United States; 8 Almost Home Kids Ann & Robert H Lurie Children’s Hospital Chicago, IL United States; 9 Franciscan Medical Specialists Munster, IN United States; 10 Department of Family Medicine and Community Health University of Minnesota Minneapolis, MN United States; 11 Judge Baker Center Harvard Medical School Roxbury Crossing, MA United States; 12 Institute for Health Research and Policy School of Public Health University of Illinois at Chicago Chicago, IL United States; 13 University of Illinois Cancer Center University of Illinois at Chicago Chicago, IL United States; 14 Department of Psychiatry University of Illinois at Chicago College of Medicine Chicago, IL United States; 15 Dr Karen Taylor-Crawford & Associates Palos Park, IL United States; 16 Department of Psychiatry Windsor University School of Medicine Cayon St Kitts Saint Kitts and Nevis

**Keywords:** adolescent, depression, prevention, scalable, eHealth

## Abstract

**Background:**

Adolescent depression carries a high burden of disease worldwide, but access to care for this population is limited. Prevention is one solution to curtail the negative consequences of adolescent depression. Internet interventions to prevent adolescent depression can overcome barriers to access, but few studies examine long-term outcomes.

**Objective:**

This study compares CATCH-IT (Competent Adulthood Transition with Cognitive Behavioral Humanistic and Interpersonal Training), an internet-based intervention, to a general health education active control for depression onset at 12 and 24 months in adolescents presenting to primary care settings.

**Methods:**

A 2-site randomized trial, blinded to the principal investigators and assessors, was conducted comparing Competent Adulthood Transition with Cognitive Behavioral Humanistic and Interpersonal Training to health education to prevent depressive episodes in 369 adolescents (193 youths were randomly assigned to Competent Adulthood Transition with Cognitive Behavioral Humanistic and Interpersonal Training and 176 to health education) with subthreshold depressive symptoms or prior depressive episodes. Participants were recruited from primary care settings in the United States. The primary outcome was the occurrence of a depressive episode, determined by the Depression Symptom Rating. The secondary outcome was functioning, measured by the Global Assessment Scale.

**Results:**

In intention-to-treat analyses, the adjusted hazard ratio favoring Competent Adulthood Transition with Cognitive Behavioral Humanistic and Interpersonal Training for first depressive episode was not statistically significant at 12 months (hazard ratio 0.77, 95% CI 0.42-1.40, *P*=.39) and 24 months (hazard ratio 0.87, 95% CI 0.52-1.47, *P*=.61). Competent Adulthood Transition with Cognitive Behavioral Humanistic and Interpersonal Training provided preventive benefit for first depressive episode for those with mild hopelessness or at least moderate paternal monitoring at baseline. Global Assessment Scale scores improved comparably in both groups (intention-to-treat).

**Conclusions:**

A technology-based intervention for adolescent depression prevention implemented in primary care did not have additional benefit at 12 or 24 months. Further research is necessary to determine whether internet interventions have long-term benefit.

**Trial Registration:**

ClinicalTrials.gov NCT01893749; http://clinicaltrials.gov/ct2/show/NCT01893749.

## Introduction

As many as 7%-13% of adolescents living in the United States and other high-income countries experience minor or major depressive episodes each year [1]. Adolescents with depressive episodes, compared to those without depression, have a higher incidence of social adjustment challenges [2,3] and are at risk for future suicide attempts and recurrent depressive episodes throughout life [4]. Unfortunately, only a minority of depressed adolescents receive treatment [5]. The negative consequences of untreated depression, delayed treatment, and extensive lack of access to treatment call for evidence-based preventive interventions that can be disseminated widely.

Recent systematic reviews and meta-analyses [6,7] found that evidence-based interventions (eg, cognitive behavioral therapy) for adolescent depression prevention and treatment could be successfully delivered via the internet [6], indicating their potential to increase access to effective preventive programs. While digital interventions can circumvent geographic barriers, time constraints, and stigma by allowing users to access interventions from their personal devices, their effects on adolescent depression prevention are mixed [7,8], and more intensive interventions with human support or guidance may have superior results [7,8].

To this point, most digital-based preventive interventions have primarily focused on short-term reduction in depressive symptoms and have not focused on how the interventions impact the risk of developing depressive episodes over time. In a review [9] examining 83 studies targeting depressive outcomes in youth, of which 8 were delivered online, only one-third (32/83) specifically focused on reducing the risk of depressive episodes rather than symptoms; small effect size reductions of depressive symptoms were found postintervention, and there was a modest absolute risk reduction of depressive episodes [9]. This limitation, in part, results from a lack of prospective studies examining the long-term outcomes necessary to detect depression onset [7,10]. A prior randomized clinical trial of CATCH-IT [11-13], the internet-based depression prevention intervention evaluated in this study, demonstrated lower depressive symptoms at 6, 12, and 30 months, relative to baseline. Despite these long-term outcomes, because randomization was based on primary care delivery models of CATCH-IT, all participants in that study received CATCH-IT rather than an active comparator.

This randomized clinical trial compared the efficacy of the CATCH-IT intervention to an online general health education program in preventing the onset of depressive episodes at 12 and 24 months in an intermediate to high-risk group of adolescents recruited from primary care settings [14]. We previously reported that at 6 months, CATCH-IT demonstrated preventive benefit against depressive episodes compared to that offered by health education for adolescents with higher depressive symptoms at baseline but not for the entire sample [15]. Given our prior 6-month results, showing significant effects for moderation based on baseline adolescent characteristics and documenting increased benefit of psychotherapy after termination (sleeper effects) [16-18], we hypothesized that a depression prevention benefit might emerge for the CATCH-IT group, and significant moderation relationships (in prespecified theory-based covariates) might be demonstrated in intention-to-treat analysis at 12 and 24 months [14].

## Methods

### Study Design and Setting

To evaluate the efficacy of CATCH-IT in a scalable setting, we used a hybrid type 1 efficacy-implementation design in which we tested the efficacy of the clinical intervention while simultaneously collecting information for large-scale implementation [19]. Focus groups tested the prototype of the system for usability and accessibility in urban minority adolescents. Adolescent participants welcomed the platform and expressed preference for an online program that is engaging and private with some face-to-face interaction to express their feelings.

The trial model was implemented in 8 major US health systems (31 primary care sites including more than 41,000 adolescents) in a population health approach (screen all youth, offer intervention, assessment, refer those in need of treatment) with over 1200 primary care staff consenting and being trained. Data were collected from urban and suburban clinics located in Boston, Massachusetts and surrounding areas, and in Chicago, Illinois and surrounding areas, including northern Indiana. Participating primary care clinics were recruited by study staff and health care providers at the study sites. Research staff worked closely with office nursing staff conducting in-person adolescent screening and primary health care providers trained in motivational interviewing [14,20].

A more detailed description of our protocol, which follows the CONSORT statement, and a detailed description of the study design and implementation process appear elsewhere [14,20]. Institutional review board approval was received from the institutional review board of record (University of Illinois at Chicago) and local institutional review boards. A data safety and monitoring board reviewed the trial and results twice per year.

### Eligibility Criteria

Eligible adolescents were aged 13 to 18 years and had either an elevated score on the Center for Epidemiologic Studies Depression (CES-D) scale [21] (8-17 on 10-item scale at screening or ≥16 on a 20-item scale at baseline, indicating clinically significant risk for depression) [22] or a prior history of a major depressive episode or dysthymia [21,23].

Adolescents were excluded if they had a current DSM-IV (Diagnostic and Statistical Manual of Mental Disorders, Fourth Edition) diagnosis of major depressive disorder or were currently being treated for major depressive disorder. Adolescents with schizophrenia, psychosis, bipolar disorder, significant reading impairment or developmental disability, current drug or alcohol abuse, imminent risk of suicide, or who were in treatment for serious medical conditions were also excluded.

### Participant Recruitment and Enrollment

**Figure 1:**
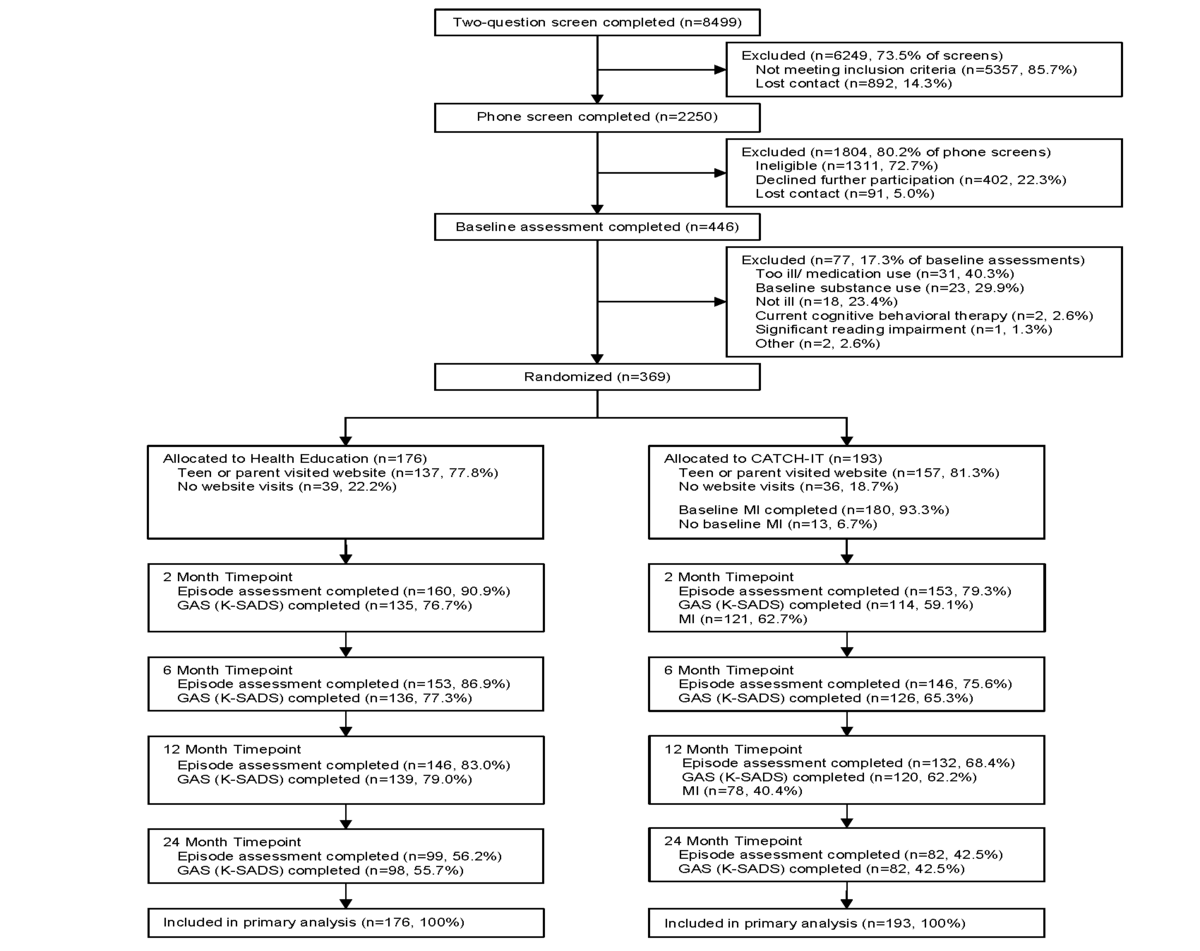
CONSORT Diagram. CATCH-IT: Competent Adulthood Transition with Cognitive Behavioral Humanistic and Interpersonal Training; GAS: Global Assessment Scale; K-SADS: Kiddie Schedule for Affective Disorders and Schizophrenia; MI: motivational interview.

Adolescents were recruited from February 2012 to July 2016 via screening, posted flyers, recruitment letters (sent to all families) and by information offered directly to families by their primary care physician during clinic visits. Initial screening for risk of depression was administered either during a face-to-face interview or over the phone using the Patient Health Questionnaire-2 [11,24]. A positive screen was the presence of any cardinal depression symptom (depressed mood, irritability, or anhedonia over the prior 2 weeks). Eligible participants and parents were then referred for an enrollment assessment at the office of their primary care physician. During this appointment, eligibility was formally confirmed through a full assessment (using Kiddie Schedule for Affective Disorders Scale, KSADS; CES-D10), accessibility to a computer and internet was confirmed, and baseline informed consent was obtained (see Figure 1 for CONSORT diagram). Participants were provided with the study website URL and personal log-in information.

### Implementation

We previously published a report [20] on the implementation of this study in 30 participating clinics with 42,310 adolescents (data were not available on adolescents from 1 additional clinic). A total of 369 adolescents were randomized into the intervention or control program. The average reach (the proportion of target audience exposed to the intervention) for screening across the Chicago study sites was 0.216, and the average reach value for enrollment across all study sites was 0.181. We describe the effect of internal barriers to recruiting adolescents (eg, screening adolescents, transferring screening results to the research team) on the screening and enrollment process by reporting reach; these internal barriers, which were within the practices, are related to the implementation of universal screening [20].

### Randomization and Masking

Random assignment to CATCH-IT or health education was achieved via a computer-generated sequence blocked by site and time of entry. Participants were also stratified according to their gender, age (13-14 years; 15-18 years) [25,26], and the severity of their risk of depressive episode (current CES-D score or prior episode of major depressive disorder). Allocation to CATCH-IT versus health education was 1:1. Although condition assignment was kept from participants, families, clinicians, and investigators until all initial assessments and preliminary data gathering were completed, it was not possible to blind the participants to their assignment, nor could the primary care clinician be blinded, as CATCH-IT implementation required clinicians to perform 3 motivational interviews over the course of the study. However, the 2 arms were presented to adolescents and parents as different approaches to improving adolescent health (eg, coping skills training or physical health improvement). Assessors were blinded for the duration of the study, and investigators were blinded until all 12-month data were collected.

### Treatment Arms

#### The CATCH-IT Intervention

CATCH-IT, an internet-based depression prevention intervention delivered in primary care settings, sought to address gaps in the field of adolescent depression prevention: (1) the implementation of a low cost and low burden intervention in primary care where youth and their families usually receive care and (2) the efficacy of an intervention in reducing diagnosed depressive episodes (versus only symptom reduction) across a 24-month follow-up period [27,28]. CATCH-IT, originally developed in the United States, incorporates cognitive behavioral therapy and interpersonal psychotherapy and was developed to have high levels of interactivity using previously published methods [27,28].

CATCH-IT integrates a set of sequential online modules with a coaching component consisting of direct interviews and periodic phone calls. There are 15 adolescent modules (14 modules on depression and an optional anxiety module with relaxation technique) designed to develop coping strategies protective against depression and 5 parent modules [29] (described in prior publications in detail) [14,15,29]. Each module takes approximately 15-20 minutes. Adapted from the Coping with Depression Adolescent Course, in combination with behavioral activation strategies (for example, behavioral scheduling of pleasurable activities) [30] and techniques used in interpersonal psychotherapy (for example, effective social problem solving, building and engaging social support) [31], CATCH-IT encourages the user to adopt a proactive approach toward mental health maintenance. Motivational support includes 3 formal motivational interviews delivered at baseline, 2 months, and 12 months by the participant’s primary care physician, and 1 to 3 coaching phone calls by study staff. A separate CATCH-IT parent program, based on Beardslee and Gladstone’s Preventive Intervention Project [32], included 4 modules that provided information about youth depression and ways to promote resilience in adolescents, and included a separate module for parents who think they may be depressed.

#### The Health Education Intervention

Health education, a 14-module online health educational tool, was used as an active control. Health education addresses adolescent health, wellness, and general safety. Module 14 discusses the identification of mood symptoms, the importance of seeking treatment for mental health concerns, and stigma associated with mental health treatment that might act as a barrier towards seeking professional help [33]; 4 modules for parents were included.

#### Shared Elements Across Both Treatment Arms

Both intervention arms provided care consistent with Guidelines for Adolescent Depression in Primary Care (GLAD-PC) [34], closely related to the chronic care model. This included screening adolescents for depression; training clinicians in the identification, diagnosis, and treatment of depression; establishing a treatment plan; creating referral relationships; and developing a safety plan [34]. Up to 3 check-in calls were made during the first 3 weeks of intervention to ensure participants had gained access to the website. We provide estimates of nonintervention time related to assessment and follow-up. These were developed based on total number of calls and sampling of call duration (40 randomly selected files to determine mean duration of calls and assessments).

### Outcomes

The primary outcome was occurrence of first depressive episode, where a Depression Symptom Rating (DSR) score indicating at least subthreshold major depression (DSR≥3) was considered a depressive episode. The secondary outcome was mental health functioning measured by GAS scores (Multimedia Appendix 1: Table S10).

### Measurements

#### Demographics

At baseline and during follow-up assessments, demographic information, educational status, and institution were collected from the participants and their families.

#### Fidelity and Adherence

Fidelity to the intervention was based on completion of the motivation interviews, quality of the motivation interviews as rated by the Motivational Interview Treatment Integrity (4.2.1) which ranges from 1 (low quality) to 5 (high quality) [35] and number of characters typed into the CATCH-IT website. We also measured modules completed and total time on the website by adolescents and parents. Additionally, at the 2-year time point, we asked 11 adolescents and parents 4 questions regarding satisfaction with the study and intervention.

#### Depressive Episodes

The 2-question screener was based on the Patient Health Questionnaire-Adolescent [24]. The K-SADS [36,37], a semistructured interview assessing current and lifetime psychiatric diagnoses in participants under age 18, was administered with parents and adolescents by licensed mental health professionals [36,38]. DSR scores were obtained from the K-SADS and the Kiddie Longitudinal Interval Follow-up Evaluation [38], which was used to identify each week of the follow-up interval’s onset and offset of diagnoses based on recall, and GAS ratings were ascertained at each assessment. The K-SADS and GAS were completed at baseline and at 2, 6, 12, and 24 months. To test for robustness of findings, we examined outcomes using DSR cut-points of ≥3, ≥4, and ≥5, indicating probable major depressive episode [39]. We selected DSR≥3 (at least subthreshold major depressive episode) versus DSR≥4 (probable or definite major depressive episode) as our primary outcome due to the lower than expected occurrence of DSR≥4 in the entire sample, identified during data safety monitoring board reviews of data. For assessment of presence of current or prior episodes (secondary versus primary prevention), we conducted an exploratory analysis of whether presence or absence of DSR≥3 or ≥4 affected subsequent outcomes.

#### Covariates in Moderation Analyses

We have proposed and published the technology-based behavioral vaccine model as an integrated conceptual framework for understanding the prevention of depression across the lifespan through internet-based interventions in community settings [10,29,40]. Based on this model, we propose moderators within this conceptualization: (1) a life course schedule that is theory-driven and that includes booster doses operationalized as patient or participant factors influencing response over the life course, including demographics such as gender, race, ethnicity, age, parental education, and site; vulnerability factors (CES-D10, Adolescent Life Events Questionnaire, Beck Hopelessness Scale, Child Report of Parental Behavior Inventory); child comorbid psychopathology (Child Anxiety Related Emotional Disorders; Disruptive Behaviors Disorder Scale; substance abuse, using CRAFFT [Car, Relax, Alone, Forget, Friends, Trouble]); and parent psychopathology (CES-D10); (2) effective components of information and training to encode responses to future threats that can then be deployed at some future points using information (operationalized as internet modules completed, time on site); (3) a motivational framework to boost response to behavior prescription (intrinsic motivation operationalized as Theory of Planned Behavior Scale, Transtheoretical Model Scale) as well as personal relevance (Sociocultural Relevance Scale); and (4) a structured implementation strategy as, to intervene effectively, we must address or provide proper conditions for successful participation in the intervention such as positive primary care relationships (operationalized Physician Relationship Scale) [11,14,30,41].

### Statistical Analysis

We estimated incidence rates by calculating the number of depressive episodes per 10,000 person-weeks of follow-up. Kaplan-Meier curves were used to estimate the time to first episode distribution for each intervention under 6 different treatment allocations: intention-to-treat, modified intention-to-treat (CATCH-IT adolescents who completed the baseline motivational interview), as treated (visited website at least once), per-protocol 2, per-protocol 4, and per-protocol 7 (adolescent and parent completed a total of 2, 4, 7 modules, respectively) (Multimedia Appendix 1: Table S1-2). Cox proportional hazard regression was used to estimate the hazard ratio comparing CATCH-IT to health education, adjusted for covariates. We present hazard ratios and coefficients adjusted for gender, ethnicity (Hispanic or non-Hispanic), race (White or non-White), baseline age, site (Boston or Chicago), and baseline CES-D10 score. We conducted exploratory analysis of the moderating effects of theory-based covariates by including interaction terms in the Cox models. We conducted the analysis of CES-D10 as a moderator in both the full sample and the subsample with elevated baseline CES-D10 scores. To examine whether the sizable number of moderator analyses we tested reveal significant effects more than by chance, we plotted all the ordered *P* values that were observed against what would be observed by chance in a quantile–quantile plot (Multimedia Appendix 1: Figure S1). We used linear mixed-effect growth models with random intercept and slope to examine differences between groups in change over time in GAS. We transformed the time scale in our growth models toward linearity by using a logarithm transformation of time. For the exploratory analysis of the impact of prior episodes on outcomes, standard descriptive statistics were used. Original sample size calculations to achieve 80% power were based on randomization of 200 into each arm and 28% incidence in the control condition at 12 months, a constant hazard ratio of 0.62, and total attrition of 36% of the sample [14]. Analyses were conducted using R (version 3.3.1), SAS (version 9.4; SAS Institute), and Mplus (version 8) [42].

## Results

### Participants

Of the 369 participants, 193 were randomized to CATCH-IT, and 176 were randomized to health education (Figure 1); depressive episode assessments (K-SADS) were available for 278 participants (75.3%) at 12 months and 181 (49.1%) at 24 months or greater (Multimedia Appendix 1: Table S3). The mean time observed was 524.8 days (SD 314.8 days) from baseline to final K-SADS. Predictors of missing episode assessment data were consistent at 12 and 24 months and included Chicago site, CATCH-IT group, older age, and lower parental education (Multimedia Appendix 1: Table S4-S5).

Adolescents were 13 to 18 years (mean 15.4 years, SD 1.5); 62% (233/3654) of adolescents had at least a prior subthreshold depressive episode (DSR≥3), and 40% (147/3654) had at least a prior probable depressive episode (DSR≥4). At baseline, the mean CES-D10 (0-30 scale) score was 9.4 (SD 4.6), the mean GAS score was 78.1 (SD 9.4), and the mean score on the Beck Hopelessness Scale was 4.7 (SD 3.6). Of the 369 participants, 16 (4.3%) adolescents reported recurrent thoughts (subthreshold or threshold) of death at baseline, 2 (0.5%) adolescents reported subthreshold suicidal ideation, and 2 (0.5%) adolescents reported subthreshold nonsuicidal self-harm thoughts. For parents at baseline, the mean CES-D10 score was 6.4 (SD 5.0), and 22.9% (72/314) were at least moderately depressed (CES-D20≥16). Adolescents were diverse in self-reported race and ethnicity: 159/369 (43.1%) identified as non-Hispanic White; 94/369 (25.5%) identified as non-Hispanic Black; and 77/369 (20.9%) identified as Hispanic. The parents of about half of the adolescents (mothers: 144/359, 40.1%; fathers: 157/336, 46.7%) had earned a high school diploma or less, and 39.4% (143/363) were not married. Relative to Chicago, the Boston site had more adolescents who identified as White (*P*<.001), a lower percentage of ethnic minority adolescents (*P*=.01), greater levels of parent education (*P*=.03 for mothers and *P*<.001 for fathers), more parents who were married at baseline (*P*=.03), and parents who were older (*P*<.001) (Multimedia Appendix 1). A complete description of the cohort data at baseline is provided in a prior publication [15].

### Fidelity and Adherence

Tests of fidelity and adherence have been described elsewhere [15] and are shown in Table 1 and Multimedia Appendix 1: Tables S11-S12. The median number of adolescent and parent modules completed was 4 in the CATCH-IT group and 8 in the health education group. Number of characters typed was used to measure active use of the modules, with adolescents and parents (combined) in the CATCH-IT arm typing a mean of 3713 (median 1899) characters. Adolescents spent more time on the CATCH-IT site than on the health education site (CATCH-IT: median 39.6 minutes; health education: median 8.4 minutes) but completed more modules on health education (CATCH-IT: median 1.0; health education: median 4.0). For CATCH-IT, 14 adolescents (14/193, 7.3%) completed all 15 modules; for health education, 74 adolescents (74/176, 42.0%) completed all 14 modules.

The median number of motivational interviews completed was 2 for both adolescents and parents, with 94.8% (183/193) of adolescents completing at least 1 and 34.2% (66/193) completing all 3 (Table 1 and Multimedia Appendix 1: Table S11). Motivational interviews were assessed for fidelity by 2 trained raters using the Motivation Interview Treatment Integrity 4.2.1 coding manual. The mean length of motivation interview was 7.7 minutes (SD 4.0), and mean lengths of technical and relational global ratings were 3.0 (SD 0.5) and 2.9 (SD 0.6), respectively (Multimedia Appendix 1: Table S11). Based on a log recording telephone and email contacts with Chicago participants (Multimedia Appendix 1: Table S12), a mean of 17.0 (SD 8.9) and 14.1 (SD 6.5) contacts were made with CATCH-IT and health education participants, respectively. We estimated mean total time required to complete assessment and follow-up common to both arms with GLAD-PC model and total time specific for the intervention (101 minutes for assessment and follow-up common to both arms, 171 minutes for CATCH-IT and 31 minutes for health education, and in total contact time, assessment and follow-up plus intervention time, 272 minutes versus 132 minutes for CATCH-IT and health education, respectively) (Multimedia Appendix 1: Table S13). With regard to the end of study satisfaction questions, adolescents noted they valued the periodic assessment calls above all other study elements, regardless of study arm.

**Table 1:** Website use, motivational interviews, and participant contacts.

Outcomes			CATCH-IT^a^, mean (SD)	Health education, mean (SD)	CATCH-IT, median (IQR)	Health education, median (IQR)	*P* value^b^
**Website use**							
	**Adolescents^c^**						
		Modules completed	3.4 (4.7)	6.8 (6.5)	1 (4)	4 (14)	.003
		Total minutes on site	100.2 (143.1)	22.8 (31.0)	39.6 (149.2)	8.4 (35.1)	<.001
		Days visited site	3.7 (4.5)	1.4 (1.6)	2 (4)	1 (2)	<.001
		Total characters typed	3071 (4572)	N/A^d^	923 (4469)	N/A	N/A
	**Parents^e^**						
		Modules completed	2.1 (2.0)	2.2 (1.9)	2 (4)	4 (4)	.80
		Total minutes on site	32.6 (37.3)	8.6 (10.0)	22.4 (51.9)	5.6 (14.9)	<.001
		Days visited site	1.6 (1.6)	0.9 (1.1)	1 (2)	1 (1)	<.001
		Total characters typed	716 (977)	N/A	101 (1205)	N/A	N/A
	**Adolescents and parents**						
		Modules completed	5.3 (5.8)	8.8 (7.3)	4 (8)	8 (17)	<.001
		Total minutes on site	130.6 (157.9)	30.6 (35.6)	75.8 (192.2)	18.9 (40.8)	<.001
		Days visited site	5.2 (5.2)	2.2 (2.2)	4 (6)	2 (2)	<.001
		Total characters typed	3713 (4932)		1899 (5792)		
**Motivational interviews^f^**							
	Adolescents		2.0 (0.9)	N/A	2 (2)	N/A	N/A
	Parents		1.9 (0.9)	N/A	2 (2)	N/A	N/A
	Adolescents and parents		3.8 (1.8)	N/A	4 (4)	N/A	N/A
Participant contacts^g^			17 (9)	N/A	18 (13)	N/A	.004

^a^CATCH-IT: Competent Adulthood Transition with Cognitive Behavioral Humanistic and Interpersonal Training.

^b^Medians were compared using Wilcoxon rank-sum test.

^c^n=193 for the CATCH-IT group; n=176 for the health education group.

^d^N/A: not applicable.

^e^n=165 for the CATCH-IT group; n=157for the health education group.

^f^CATCH-IT adolescents and parents were offered 3 motivational interviews; see Multimedia Appendix 1 for additional information.

^g^n=248 telephone calls and emails with Chicago participants, including administrative contacts such as scheduling calls in addition to coaching calls, safety checks, and motivational interview calls. See Multimedia Appendix 1 for additional information.

### Time to Depressive Episode

In intention-to-treat analyses, the hazard ratio favored CATCH-IT for occurrence of first depressive episode but was statistically significant neither at 12 months (hazard ratio 0.77, 95% CI 0.42-1.40, *P*=.39; Multimedia Appendix 1: Table S1) nor at 24 months (hazard ratio 0.87, 95% CI 0.52-1.47, *P*=.61, Multimedia Appendix 1: Table S2). For per-protocol 2 analysis (at least 2 modules completed by adolescent and parent combined in either arm), the effect of CATCH-IT on depressive episode prevention was improved but remained nonsignificant at 12 months (hazard ratio 0.65, 95% CI 0.34-1.23, *P*=.18, Multimedia Appendix 1: Table S1) and 24 months (hazard ratio 0.73, 95% CI 0.41-1.28, *P*=.27; Multimedia Appendix 1: Table S2). Annual incidence of first at least subthreshold depressive episode (DSR≥3) was 12.7% for CATCH-IT and 13.8% for health education; incidence of probable major depressive episode (DSR≥4) was 4.6% for CATCH-IT and 5.8% for health education (Multimedia Appendix 1: Table S6).

### Functional Status Improvement

GAS scores improved in each group from baseline to 24 months (Multimedia Appendix 1: Table S17). For GAS with time logarithm transformed, the slope was 2.964 (SE 0.285; *P*<.001), and adjusted change from baseline to 24 months was 9.5 (SE 0.9) for CATCH-IT, compared to a slope of 3.076 (SE 0.278; *P*<.001) and change of 9.9 (SE 0.9) for health education. There was no significant difference in slopes between groups (*P*=.78) (Multimedia Appendix 1: Table S17).

### Moderation Analyses

**Figure 2:**
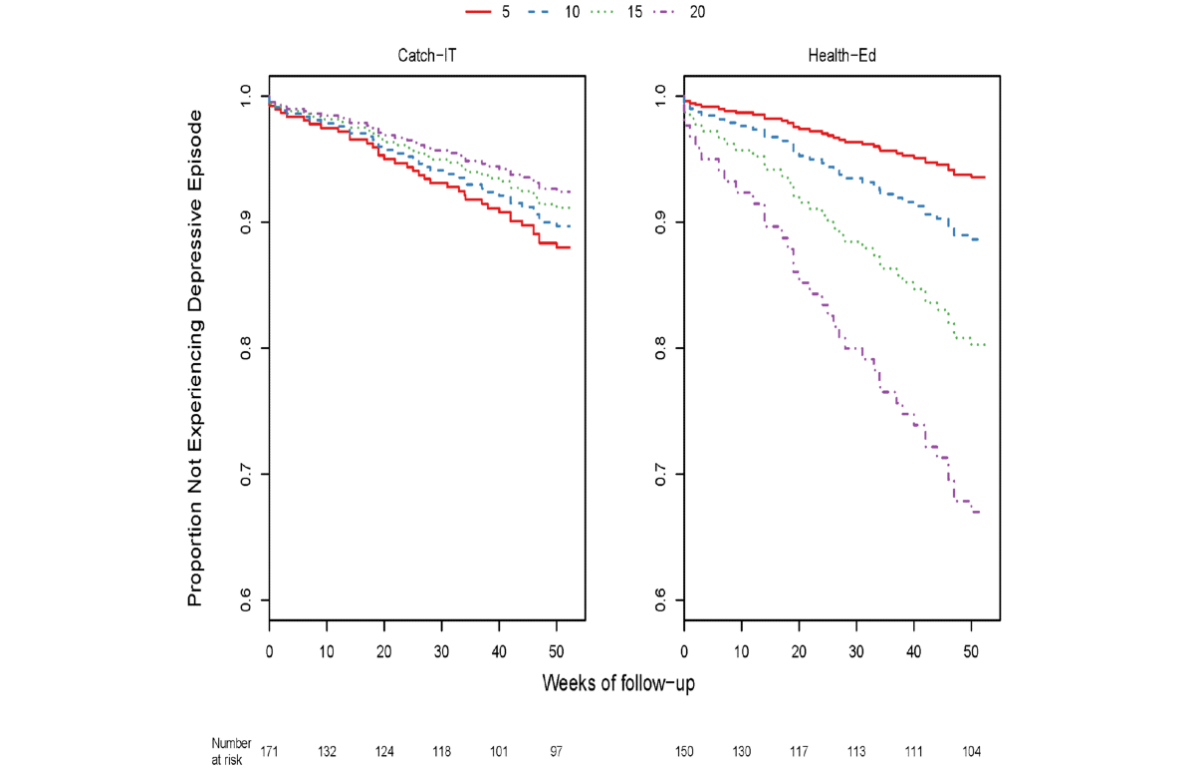
Adolescent CES-D Scale score at baseline and time to Depression Symptom Rating>3–event at 12 months, excluding those who enrolled with no depressed mood.

**Figure 3:**
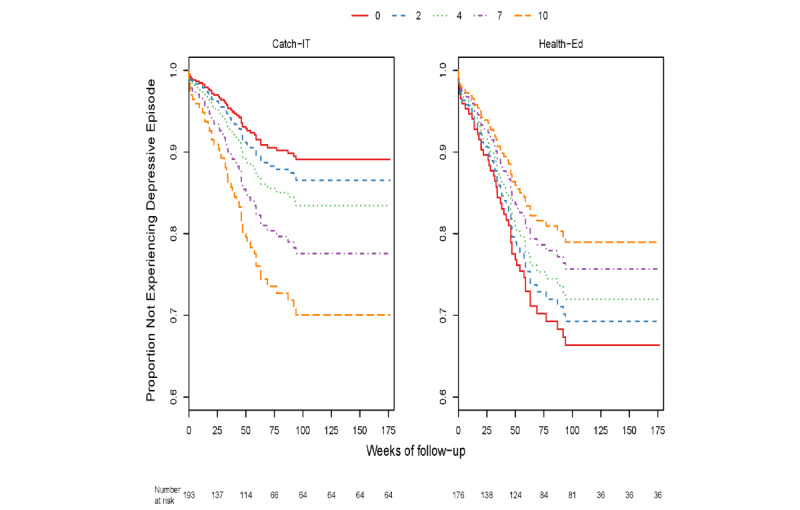
Adolescent Beck Hopelessness Scale score at at baseline and time to Depression Symptom Rating>3–event at 24 months.

**Figure 4:**
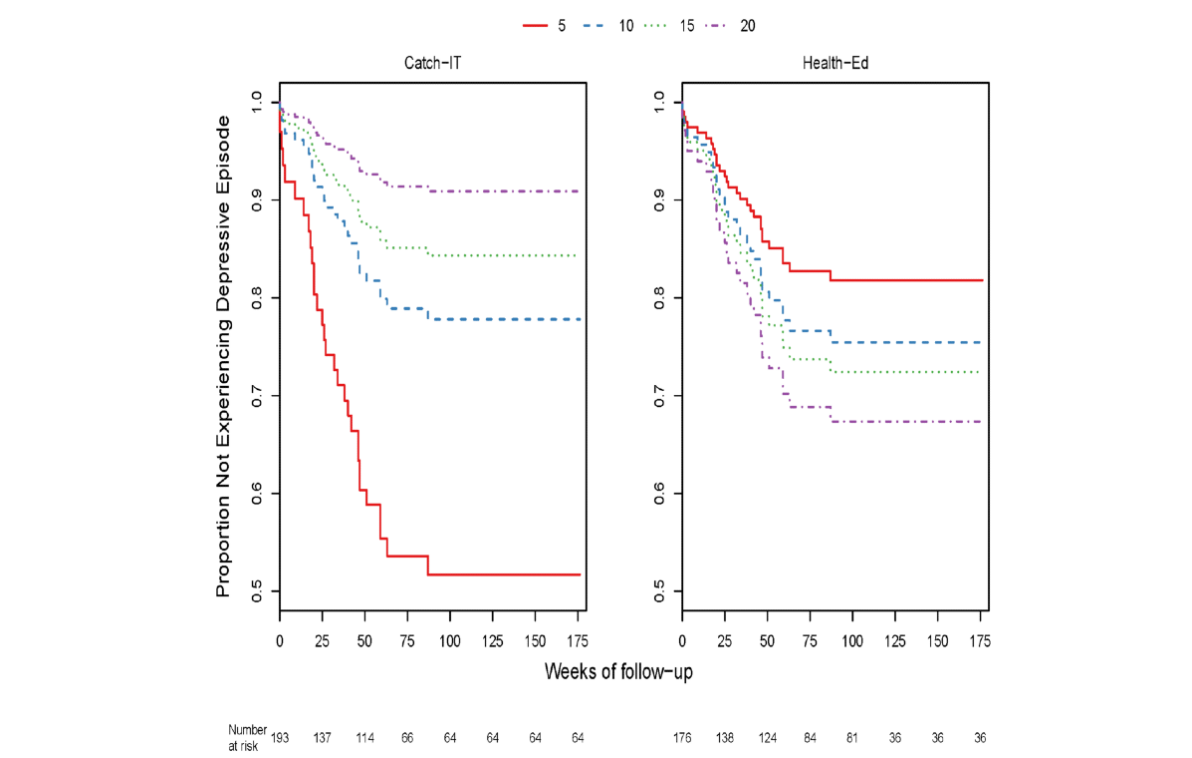
Adolescent Child Report of Parental Behaviour Inventory (Father Monitoring Subscale) score at baseline and time to Depression Symptom Rating>3–event at 24 months.

Through 12 months, within the group of adolescents who enrolled based on elevated CES-D (321/369, 86.9%) with or without a prior depressive episode, those with higher baseline CES-D10 scores exhibited a marginally stronger preventive effect of CATCH-IT on time to event (DSR≥3) relative to those with lower baseline scores (0.15 decline in the logarithm of hazard ratio for each CES-D10 point increase, *P*=.054) (Multimedia Appendix 1: Table S7, Figure 2). Those who also visited the website at least once (as treated) (253/369, 68.6%) showed a similar effect (0.18 decline in the logarithm of hazard ratio for each CES-D10 point increase, *P*=.03). Baseline CES-D10 was no longer a significant moderator at 24 months, either in the sample as a whole or in the subgroups tested. However, 2 additional moderators were identified as significant on time to event through 24 months (DSR≥3). Specifically, there was a complex interaction between baseline hopelessness (270/369, 73.2%) whereby, at lower Beck Hopelessness Scale scores, CATCH-IT demonstrated a preventive effect, but results favored health education at higher scores (*P*=.04) (Multimedia Appendix 1: Table S8, Figure 3). Similarly, there was a complex interaction between baseline paternal monitoring (170/369, 46.1%) and group assignment, such that at higher levels of paternal monitoring at baseline, CATCH-IT demonstrated preventive effects on time to event (DSR≥3) but favored health education at lower paternal monitoring (*P*=.048, Multimedia Appendix 1: Table S9, Figure 4). Site, race, ethnicity, and gender did not moderate outcomes. We conducted an overall comparison of the 12-month and 24-month moderation effects to see whether there was any signal evident beyond what one would expect from a null distribution. Multimedia Appendix 1: Figure S1 provides a comparison of all the ordered *P* values against this standard. The moderation effects for hopelessness and paternal monitoring did not stand out against the null distribution. No GAS moderation analyses yielded statistically significant results after time transformation (all *P*>.05 after logarithm transformation, Multimedia Appendix 1: Table S18).

Assessment of presence of current or prior episodes (secondary versus primary prevention) showed there was no meaningful variation in the presence of current or past DSR≥3 or ≥4 across groups at baseline (Multimedia Appendix 1: Table S14). With the main outcome threshold of DSR≥3, about two-thirds of the sample could be characterized as secondary prevention and one-third primary prevention (Multimedia Appendix 1: Table S14). However, most of the episodes of DSR≥3 during follow-up were new episodes; only 4 episodes were present or persisting at 2 months in each study arm (Multimedia Appendix 1: Table S15). Adolescents with past or current episodes at baseline were statistically equally likely to have a follow-up episode regardless of group assignment. For adolescents with a current or past episode with DSR≥3 at baseline, 17.9% of CATCH-IT and 19.8% of health education participants had an episode during the follow-up period (Multimedia Appendix 1: Table S16).

## Discussion

This hybrid Type 1 effectiveness-implementation clinical trial [19] conducted in primary care practice settings (31 sites) with a diverse sample is the only study, to our knowledge, with over 12 months of follow-up for adolescents enrolled in an internet-based study [13,43,44]. At 12 and 24 months postintervention using Cox proportional hazard regression analysis, we found that CATCH-IT was not superior to health education in preventing the onset of depressive episodes. The annual incidence of major depressive episodes was fairly low for both groups (DSR≥4: 4.6% CATCH-IT, 5.8% health education; DSR≥3: 13.7% CATCH-IT, 12.7%). This compares favorably with the 12-month incidence of major depressive episodes (DSR≥4) in the United States, which is 8.3% [1] in general samples and >15% in similar risk–adjusted samples; these rates are roughly half the rates for minor plus major depressive episodes in high-risk samples (DSR≥3) [1,21,23]. Moderated analyses suggested that adolescents who enroll with higher CES-D scores, lower levels of hopelessness, and higher paternal monitoring may obtain preventive benefit from CATCH-IT for 12 months, and possibly for as long as two years postenrollment.

There are several potential explanations for why this trial failed to show a main effect of the CATCH-IT intervention. First, the incidence of depressive episodes was much lower than anticipated for this high-risk sample, and remained lower than expected even after lowering the threshold of primary outcome from probable depressive episode (DSR ≥4) to subthreshold depressive episode (DSR≥3). An important factor may be the amount of assessment follow-up time common to both arms (150 minutes) within the framework of the GLAD-PC guidelines [36], which was nearly equal to the total intervention time for CATCH-IT (170 minutes), rather than any particular effect of the health education intervention (30 minutes). It is possible that this level of assessment and follow-up suppressed the level of depressive episodes in both arms, depriving the CATCH-IT intervention of some of the potential to prevent episodes. The demonstrated intervention of a Chinese language CATCH-IT at 12 months which had only self-assessments but did have an attention control is consistent with this explanation [45], as is the significant improvement in functional status over time in both groups. Another explanation would be that, in fact, the active control reduced between group differences since most adolescent studies with active controls have not demonstrated between group differences [46,47]. The recruitment of slightly less than the target sample size (92%), higher attrition (>50% versus 36% anticipated), weaker intervention effect (hazard ratio 0.87 versus predicted hazard ratio 0.62), and lower dose (hazard ratio more favorable for those with higher dose) could all play roles in the nonsignificant results in intention-to-treat analyses. Perhaps a dose threshold of 2-4 modules (including cognitive restructuring, behavioral activation) is essential, as occurred in the Chinese CATCH-IT trial [45] and other studies [48-50]. Additionally, the fact that some teens seem to respond more favorably to the health education intervention (those with no current symptoms), while others to CATCH-IT (those with symptoms), reduced between group differences.

Many have called for examination of moderators for treatment tailoring and to contribute to future meta-analyses parsing out differential treatment outcomes [51]. Moderated results suggest that CATCH-IT may benefit a significant portion of youth, such as groups of youth with elevated depressive symptoms. For example, adolescents who were enrolled with no current depressed mood but with a prior depressive episode fared worse with CATCH-IT, while those with depressed mood may benefit for as long as 12 months. Similarly, adolescents with lower hopelessness may benefit from CATCH-IT, while those with higher levels may not benefit from such cognitive behavioral therapy–based interventions [52]. It is possible that the context of social support may matter, such that CATCH-IT, which requires youth effort (eg, completion of cognitive behavioral therapy homework), may offer preventive benefit for those who report adequate paternal monitoring, consistent with Mohr’s supportive accountability model [8]. One might speculate that more paternal involvement in the health education intervention may be overbearing because less accountability is needed. While female gender predicted higher risk of episodes, results did not vary by gender nor did they vary by ethnicity. The results of this trial along with those from prior trials [7] suggest that interventions can prevent depressive episode onset and worsening of depressive symptoms in youth subpopulations.

This study has important limitations and strengths. While it lacked an inactive or wait-list control, wait-list control is less ethical, as effective interventions are available. We cannot deconstruct the effects of motivation interview versus the online modules, but depressive symptoms decreased for adolescents who used CATCH-IT even without a motivation interview in our pilot study [12]. Differential loss to follow-up for those in the CATCH-IT group and at the Chicago site is concerning. However, predictors of increased risk of episodes, such as depressed mood or female gender, did not predict loss to follow-up, and make it unlikely that hazard ratios or incidence of episodes were affected by dropout patterns. Measurement of depressive episodes is challenging, and measurement issues could explain lower than expected rates. However, both sites were trained by an experienced evaluation team with methods used in prior clinical trials [39]. Despite these limitations, this study has several strengths. The standardized implementation across several primary care settings supports feasibility of this model of care, though it will be important to determine potential logistical barriers such as check-in calls to participants. The examination of depressive episodes, long-term follow up, active control, and blinding of outcome assessors address methodological limitations of prior studies.

Future investigators should exercise caution in the design of low-intensity technology-based preventive interventions when considering active controls and extensive assessment batteries. Rather than an active control, an informational brochure may be a better option for a control; likewise, rather than using the KSADS to gather diagnostic information, the much shorter Mini-International Neuropsychiatric Interview for Children and Adolescents could be considered. Similarly, careful stakeholder-grounded intervention design (additional incentives or complementary design features, for example, online discussion groups or animation) may be needed to induce adolescent-parent pairs to complete more modules and thus strengthen the preventive effect to ensure minimum dose levels in the target population. Moderated results suggest that life course factors (depressed mood) and family factors (paternal monitoring) may be related to the potential for efficacy beyond 6 months. For example, a general health promotion model intervention may be more appropriate for adolescents at risk for a major depressive episode but currently with minimal symptoms, or conversely, CATCH-IT may not benefit adolescents with high levels of hopelessness and low levels of paternal monitoring. In short, we have a great deal to learn about how to implement technology-based depression prevention interventions and for whom they will work, in primary care and community settings. Future research should investigate how to best tailor online interventions to the characteristics of teens at risk of depression in order to optimize outcomes and prevent the development of major depression.

## Appendix

Multimedia Appendix 1 Supplemental tables and figure.

## Abbreviations

CATCH-IT: Competent Adulthood Transition with Cognitive Behavioral Humanistic and Interpersonal Training
CES-D: Center for Epidemiologic Studies - Depression
CONSORT: Consolidated Standards of Reporting Trials
DSR: Depression Symptom Rating
GAS: Global Assessment Scale
GLAD-PC: Guidelines for Adolescent Depression in Primary Care
K-SADS: Kiddie Schedule for Affective Disorders Scale
