# Supplementary material for: 24-Month Outcomes of Primary Care Web-Based Depression Prevention Intervention in Adolescents: Randomized Clinical Trial
Source: J Med Internet Res. 2020 Oct 28;22(10):e16802. doi: 10.2196/16802 (PMC7657722; doi:10.2196/16802)
Supplement: Multimedia Appendix 1 [file jmir_v22i10e16802_app1.docx]

**Appendix**

**Table S1. Hazard Ratio Estimate and 95% CI for First Depressive Episode Comparing Catch-It to Health Education, 12-month Cut-point**

|  |  | **Unadjusted** | | | |  | **Adjusted*** | | | |
| --- | --- | --- | --- | --- | --- | --- | --- | --- | --- | --- |
|  |  | **HR** | **95% CI** | | ***P*-value** |  | **HR** | **95% CI** | | ***P*-value** |
| **Intent to Treat  (N = 369)** | **DSR >= 3** | 0.83 | (0.47, | 1.48) | .53 |  | 0.77 | (0.42, | 1.40) | .39 |
|  | **DSR >= 4** | 0.78 | (0.33, | 1.85) | .58 |  | 0.82 | (0.34, | 1.97) | .65 |
|  | **DSR = 5** | 0.60 | (0.18, | 2.06) | .42 |  | 0.59 | (0.17, | 2.01) | .40 |
| **Modified Intent to Treat  (N = 356)** | **DSR >= 3** | 0.86 | (0.48, | 1.53) | .61 |  | 0.81 | (0.45, | 1.48) | .50 |
|  | **DSR >= 4** | 0.81 | (0.34, | 1.91) | .62 |  | 0.86 | (0.36, | 2.09) | .75 |
|  | **DSR = 5** | 0.62 | (0.18, | 2.12) | .45 |  | 0.61 | (0.18, | 2.10) | .44 |
| **As Treated  (N = 294)** | **DSR >= 3** | 0.80 | (0.45, | 1.44) | .46 |  | 0.75 | (0.41, | 1.37) | .34 |
|  | **DSR >= 4** | 0.73 | (0.31, | 1.73) | .47 |  | 0.78 | (0.32, | 1.89) | .58 |
|  | **DSR = 5** | 0.56 | (0.17, | 1.93) | .36 |  | 0.57 | (0.17, | 1.95) | .37 |
| **Per Protocol 2  (N = 245)** | **DSR >= 3** | 0.65 | (0.34, | 1.21) | .17 |  | 0.65 | (0.34, | 1.23) | .18 |
|  | **DSR >= 4** | 0.69 | (0.28, | 1.70) | .42 |  | 0.75 | (0.30, | 1.88) | .54 |
|  | **DSR = 5** | 0.60 | (0.18, | 2.06) | .42 |  | 0.62 | (0.18, | 2.12) | .44 |
| **Per Protocol 4  (N = 222)** | **DSR >= 3** | 0.60 | (0.31, | 1.18) | .14 |  | 0.59 | (0.30, | 1.19) | .14 |
|  | **DSR >= 4** | 0.67 | (0.26, | 1.69) | .39 |  | 0.74 | (0.28, | 1.91) | .53 |
|  | **DSR = 5** | 0.66 | (0.19, | 2.27) | .51 |  | 0.72 | (0.21, | 2.52) | .61 |
| **Per Protocol 7  (N = 152)** | **DSR >= 3** | 0.68 | (0.28, | 1.65) | .40 |  | 0.62 | (0.24, | 1.58) | .32 |
|  | **DSR >= 4** | 0.68 | (0.17, | 2.61) | .57 |  | 0.76 | (0.18, | 3.16) | .70 |
|  | **DSR = 5** | 0.80 | (0.15, | 4.35) | .79 |  | 2.73 | (0.24, | 30.52) | .42 |

*Note*: * Adjusted for gender, race (white vs. non-white), ethnicity (Hispanic vs. non-Hispanic), age at baseline, site, and baseline CES-D_10_ score

**Table S2. Hazard Ratio Estimate and 95% CI for First Depressive Episode Comparing Catch-It to Health Education, 24-month Cut-point**

|  |  | **Unadjusted** | | | |  | **Adjusted*** | | | |
| --- | --- | --- | --- | --- | --- | --- | --- | --- | --- | --- |
|  |  | **HR** | **95% CI** | | ***P*-value** |  | **HR** | **95% CI** | | ***P*-value** |
| **Intent to Treat  (N = 369)** | **DSR >= 3** | 0.90 | (0.54, | 1.49) | .68 |  | 0.87 | (0.52, | 1.47) | .61 |
|  | **DSR >= 4** | 0.77 | (0.35, | 1.67) | .50 |  | 0.80 | (0.36, | 1.76) | .57 |
|  | **DSR = 5** | 0.60 | (0.18, | 2.06) | .42 |  | 0.59 | (0.17, | 2.01) | .40 |
| **Modified Intent to Treat  (N = 356)** | **DSR >= 3** | 0.93 | (0.56, | 1.54) | .78 |  | 0.91 | (0.54, | 1.53) | .72 |
|  | **DSR >= 4** | 0.79 | (0.36, | 1.72) | .56 |  | 0.84 | (0.38, | 1.85) | .66 |
|  | **DSR = 5** | 0.62 | (0.18, | 2.12) | .45 |  | 0.61 | (0.18, | 2.10) | .44 |
| **As Treated  (N = 294)** | **DSR >= 3** | 0.86 | (0.51, | 1.44) | .57 |  | 0.84 | (0.49, | 1.44) | .53 |
|  | **DSR >= 4** | 0.77 | (0.35, | 1.70) | .52 |  | 0.85 | (0.38, | 1.89) | .68 |
|  | **DSR = 5** | 0.56 | (0.17, | 1.93) | .36 |  | 0.57 | (0.17, | 1.95) | .37 |
| **Per Protocol 2  (N = 245)** | **DSR >= 3** | 0.71 | (0.41, | 1.23) | .22 |  | 0.73 | (0.41, | 1.28) | .27 |
|  | **DSR >= 4** | 0.68 | (0.29, | 1.56) | .36 |  | 0.73 | (0.31, | 1.72) | .48 |
|  | **DSR = 5** | 0.60 | (0.18, | 2.06) | .42 |  | 0.62 | (0.18, | 2.12) | .44 |
| **Per Protocol 4  (N = 222)** | **DSR >= 3** | 0.67 | (0.37, | 1.21) | .18 |  | 0.70 | (0.38, | 1.29) | .25 |
|  | **DSR >= 4** | 0.66 | (0.28, | 1.57) | .35 |  | 0.74 | (0.31, | 1.79) | .50 |
|  | **DSR = 5** | 0.66 | (0.19, | 2.27) | .51 |  | 0.72 | (0.21, | 2.52) | .61 |
| **Per Protocol 7  (N = 152)** | **DSR >= 3** | 0.70 | (0.32, | 1.54) | .38 |  | 0.73 | (0.32, | 1.66) | .45 |
|  | **DSR >= 4** | 0.59 | (0.16, | 2.23) | .44 |  | 0.65 | (0.16, | 2.61) | .54 |
|  | **DSR = 5** | 0.80 | (0.15, | 4.35) | .79 |  | 2.73 | (0.24, | 30.52) | .42 |

*Note*: * Adjusted for gender, race (white vs. non-white), ethnicity (Hispanic vs. non-Hispanic), age at baseline, site, and baseline CES-D 10 score

**Figure S1. Quantile-Quantile Plot of Ordered P-Values from Moderator Analyses against Values Expected by Chance**


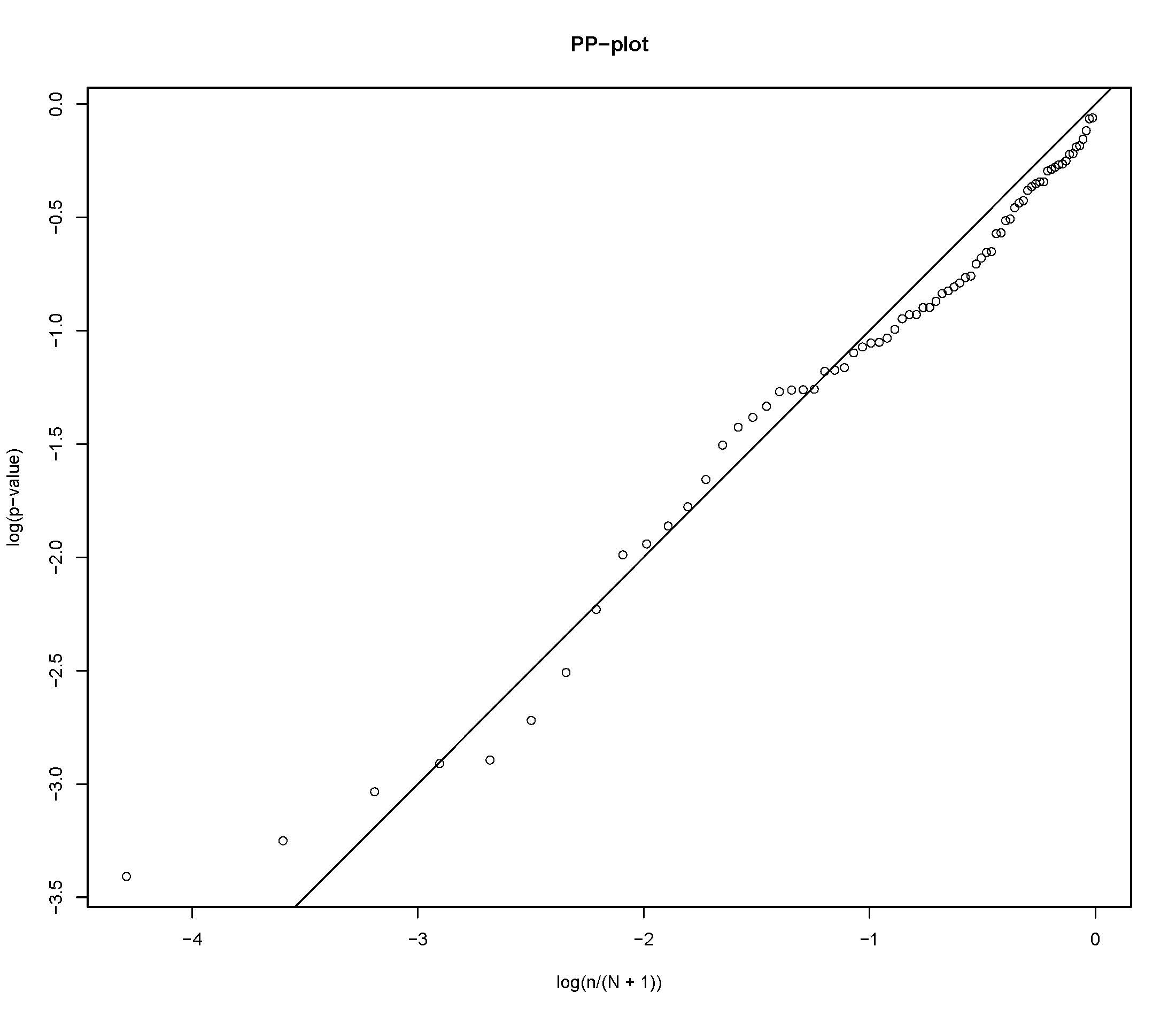


**Table S3. Time to Last Follow-up, by Group and Site**

|  | **All** | | **CATCH-IT** | | **Health Education** | |  |
| --- | --- | --- | --- | --- | --- | --- | --- |
|  | **N** | **(%)** | **N** | **(%)** | **N** | **(%)** | ***P*^a^** |
| **All Participants** | N=369 |  | N=193 |  | N=176 |  |  |
| **Time to last follow-up^b^** |  |  |  |  |  |  | .001 |
| < 12 months | 91 | (24.7%) | 61 | (31.6%) | 30 | (17.0%) |  |
| ≥ 12 months | 278 | (75.3%) | 132 | (68.4%) | 146 | (83.0%) |  |
|  |  |  |  |  |  |  |  |
| **Time to last follow-up** |  |  |  |  |  |  | .008 |
| < 24 months | 188 | (50.9%) | 111 | (57.5%) | 77 | (43.8%) |  |
| ≥ 24 months | 181 | (49.1%) | 82 | (42.5%) | 99 | (56.2%) |  |
| **Chicago** | N=248 |  | N=131 |  | N=117 |  |  |
| **Time to last follow-up** |  |  |  |  |  |  | .004 |
| < 12 months | 80 | (32.3%) | 53 | (40.5%) | 27 | (23.1%) |  |
| ≥ 12 months | 168 | (67.7%) | 78 | (59.5%) | 90 | (76.9%) |  |
|  |  |  |  |  |  |  |  |
| **Time to last follow-up** |  |  |  |  |  |  | .008 |
| < 24 months | 151 | (60.9%) | 90 | (68.7%) | 61 | (52.1%) |  |
| ≥ 24 months | 97 | (39.1%) | 41 | (31.3%) | 56 | (47.9%) |  |
| **Boston** | N=121 |  | N=62 |  | N=59 |  | .13 |
| **Time to last follow-up** |  |  |  |  |  |  |  |
| < 12 months | 11 | (9.1%) | 8 | (12.9%) | 3 | (5.1%) |  |
| ≥ 12 months | 110 | (90.9%) | 54 | (87.1%) | 56 | (94.9%) |  |
|  |  |  |  |  |  |  |  |
| **Time to last follow-up** |  |  |  |  |  |  | .42 |
| < 24 months | 37 | (30.6%) | 21 | (33.9%) | 16 | (27.1%) |  |
| ≥ 24 months | 84 | (69.4%) | 41 | (66.1%) | 43 | (72.9%) |  |
| ^a^ From chi-square test for difference between CATCH-IT and HealthEd.  ^b^ All available follow-up assessments (KSADS) were used to calculate time observed. | | | | | | | |

**Table S4. Predictors^a^ of missing 12-month episode assessment data: time to last KSADS assessment < 12 months, all participants**

|  | **OR** | **(CI)** | | **b** | ***P*** |
| --- | --- | --- | --- | --- | --- |
| Intercept | -- |  |  | -6.268 | <.001 |
| Boston (ref=Chicago) | 0.216 | (0.103 | 0.452) | -1.534 | <.001 |
| CATCH-IT (ref=HealthEd) | 2.984 | (1.682 | 5.294) | 1.093 | <.001 |
| Age at baseline, y | 1.295 | (1.085 | 1.546) | 0.259 | .004 |
| Male (ref=female) | 1.670 | (0.943 | 2.955) | 0.513 | .08 |
| Hispanic (ref=non-Hisp) | 0.939 | (0.477 | 1.850) | -0.063 | .86 |
| Non-white (ref=white) | 1.212 | (0.674 | 2.178) | 0.192 | .52 |
| Mother’s education (ref=college graduate) |  |  |  |  |  |
| HS graduate/GED or less | 5.111 | (2.411 | 10.832) | 1.631 | <.001 |
| Some college | 1.152 | (0.567 | 2.343) | 0.142 | .70 |
| Parents’ marital status (ref=married) |  |  |  |  |  |
| Never married | 0.892 | (0.399 | 1.996) | -0.114 | .78 |
| Divorced, separated, widowed | 0.696 | (0.340 | 1.427) | -0.362 | .32 |
| Firstborn child (ref=not firstborn) | 1.379 | (0.790 | 2.405) | 0.321 | .26 |
| Past episode at baseline (ref=no past episode) | 0.939 | (0.512 | 1.720) | -0.063 | .84 |
| High CESD at screening or baseline (ref=no high CESD) | 1.355 | (0.517 | 3.551) | 0.304 | .54 |
| ^a^ From logistic regression model. Some observations were excluded from the model due to missing data for covariates: N=353. | | | | | |

**Table S5. Predictors^a^ of missing 24-month episode assessment data: time to last KSADS assessment < 24 months, all participants**

|  | **OR** | **(CI)** | | **b** | ***P*** |
| --- | --- | --- | --- | --- | --- |
| Intercept | -- |  |  | -4.098 | .002 |
| Boston (ref=Chicago) | 0.270 | (0.159 | 0.458) | -1.310 | <.001 |
| CATCH-IT (ref=HealthEd) | 1.966 | (1.232 | 3.136) | 0.676 | .005 |
| Age at baseline, y | 1.274 | (1.089 | 1.491) | 0.242 | .003 |
| Male (ref=female) | 1.211 | (0.734 | 1.997) | 0.191 | .45 |
| Hispanic (ref=non-Hisp) | 1.108 | (0.605 | 2.029) | 0.102 | .74 |
| Non-white (ref=white) | 0.905 | (0.544 | 1.506) | -0.100 | .70 |
| Mother’s education (ref=college graduate) |  |  |  |  |  |
| HS graduate/GED or less | 2.572 | (1.255 | 5.269) | 0.945 | .01 |
| Some college | 0.881 | (0.490 | 1.582) | -0.127 | .67 |
| Parents’ marital status (ref=married) |  |  |  |  |  |
| Never married | 0.928 | (0.458 | 1.881) | -0.075 | .84 |
| Divorced, separated, widowed | 0.711 | (0.391 | 1.292) | -0.341 | .26 |
| Firstborn child (ref=not firstborn) | 1.483 | (0.922 | 2.386) | 0.394 | .10 |
| Past episode at baseline (ref=no past episode) | 0.748 | (0.447 | 1.252) | -0.291 | .27 |
| High CESD at screening or baseline (ref=no high CESD) | 1.520 | (0.706 | 3.272) | 0.419 | .28 |
| ^a^ From logistic regression model. Some observations were excluded from the model due to missing data for covariates: N=353. | | | | | |

**Table S6. Incidence of First Depressive Episode by Treatment Group, 24-month Cut-point**

|  |  | **Health Education** | | | **CATCH-IT** | | |
| --- | --- | --- | --- | --- | --- | --- | --- |
|  |  | **N** | **IR** | **Annual Incidence** | **N** | **IR** | **Annual Incidence** |
| **Intent to Treat  (N = 369)** | DSR >= 3 | 176 | 26.44 | 13.75 | 193 | 24.34 | 12.66 |
|  | DSR >= 4 | 176 | 11.23 | 5.84 | 193 | 8.75 | 4.55 |
|  | DSR = 5 | 176 | 5.05 | 2.62 | 193 | 3.09 | 1.61 |
| **Modified Intent to Treat  (N = 356)** | DSR >= 3 | 176 | 26.44 | 13.75 | 180 | 25.38 | 13.20 |
|  | DSR >= 4 | 176 | 11.23 | 5.84 | 180 | 9.09 | 4.73 |
|  | DSR = 5 | 176 | 5.05 | 2.62 | 180 | 3.21 | 1.67 |
| **As Treated  (N = 294)** | DSR >= 3 | 137 | 29.84 | 15.52 | 157 | 26.37 | 13.71 |
|  | DSR >= 4 | 137 | 12.43 | 6.46 | 157 | 9.78 | 5.08 |
|  | DSR = 5 | 137 | 5.96 | 3.10 | 157 | 3.45 | 1.79 |
| **Per Protocol 2  (N = 245)** | DSR >= 3 | 123 | 33.17 | 17.25 | 122 | 24.13 | 12.55 |
|  | DSR >= 4 | 123 | 13.70 | 7.12 | 122 | 9.58 | 4.98 |
|  | DSR = 5 | 123 | 6.54 | 3.40 | 122 | 4.12 | 2.14 |
| **Per Protocol 4  (N = 222)** | DSR >= 3 | 118 | 32.48 | 16.89 | 104 | 21.98 | 11.43 |
|  | DSR >= 4 | 118 | 14.35 | 7.46 | 104 | 9.65 | 5.02 |
|  | DSR = 5 | 118 | 6.83 | 3.55 | 104 | 4.70 | 2.44 |
| **Per Protocol 7  (N = 152)** | DSR >= 3 | 93 | 27.34 | 14.22 | 59 | 18.94 | 9.85 |
|  | DSR >= 4 | 93 | 10.07 | 5.24 | 59 | 5.80 | 3.01 |
|  | DSR = 5 | 93 | 4.84 | 2.52 | 59 | 3.79 | 1.97 |

*Note*: Incidence rate (IR); events per 10,000 person-weeks

**Table S7. Adjusted Cox Proportional Hazard Model for Occurrence of First Depressed Episode (DSR ≥ 3+), Including Adolescent Baseline CES-D_10_ as a Moderator, Among Those with Elevated Depressed Mood at Screening or Baseline (N=321) at 12 months**

| **Model Estimates** |  | **Beta** | **HR** | **95% CI** | | ***P*-value** |
| --- | --- | --- | --- | --- | --- | --- |
| CATCH-IT vs. HE |  | 1.42 | 4.13 | (0.68, | 25.04) | .12 |
| Male vs. Female |  | -1.41 | 0.24 | (0.09, | 0.69) | .008 |
| Non-white vs. white |  | 0.45 | 1.57 | (0.81, | 3.07) | .18 |
| Hispanic vs. not |  | 0.34 | 1.40 | (0.67, | 2.93) | .37 |
| Age at baseline |  | -0.10 | 0.90 | (0.71, | 1.15) | .41 |
| Site (Boston vs. Chicago) |  | 0.70 | 2.02 | (1.02, | 4.01) | .045 |
| Baseline CES-D |  | 0.12 | 1.13 | (1.01, | 1.26) | .04 |
| CATCH-IT * CES-D interaction |  | -0.15 | 0.86 | (0.74, | 1.00) | .05 |
| **Between Group Comparisons, comparing CATCH-IT to HE, for increasing CES-D scores** | **CES-D score** | **Beta** | **HR** | **95% CI** | | ***P*-value** |
|  | 5 | 0.66 | 1.93 | (0.63, | 5.92) | .25 |
|  | 10 | -0.10 | 0.90 | (0.46, | 1.78) | .77 |
|  | 15 | -0.86 | 0.42 | (0.17, | 1.07) | .07 |
|  | 20 | -1.63 | 0.20 | (0.04, | 0.95) | .04 |
| **Within Group Comparison, describing a one-unit increase in CES-D score** | **CES-D score** | **Beta** | **HR** | **95% CI** | | ***P*-value** |
| CATCH-IT | 1 unit change | -0.03 | 0.97 | (0.87, | 1.08) | .55 |
| HE | 1 unit change | 0.12 | 1.13 | (1.01, | 1.26) | .04 |

*Note*: Higher CES-D scores indicate higher levels of depression

*Note*: n=7 subjects missing baseline CES-D

**Table S8. Adjusted Cox Proportional Hazard Model for Occurrence of First Depressed Episode (DSR ≥ 3+), Including Beck Hopelessness as a Moderator, Full Sample (N=369) at 24 months**

| **Model Estimates** |  | **Beta** | **HR** | **95% CI** | | ***P*-value** |
| --- | --- | --- | --- | --- | --- | --- |
| CATCH-IT vs. HE |  | -1.27 | 0.28 | (0.09, | 0.83) | .02 |
| Male vs. Female |  | -0.99 | 0.37 | (0.16, | 0.84) | .02 |
| Non-white vs. white |  | 0.26 | 1.29 | (0.71, | 2.37) | .40 |
| Hispanic vs. not |  | 0.34 | 1.40 | (0.72, | 2.75) | .32 |
| Age at baseline |  | 0.00 | 1.00 | (0.81, | 1.24) | >.99 |
| Site (Boston vs. Chicago) |  | -0.01 | 0.99 | (0.54, | 1.84) | .98 |
| Baseline CES-D |  | 0.07 | 1.08 | (1.00, | 1.15) | .04 |
| Beck Hopelessness |  | -0.06 | 0.95 | (0.85, | 1.06) | .32 |
| CATCH-IT * Beck Hopelessness Interaction |  | 0.17 | 1.18 | (1.01, | 1.39) | .04 |
| **Between Group Comparisons, comparing CATCH-IT to HE, for increasing values on Beck Hopelessness** | **Beck Hopelessness** | **Beta** | **HR** | **95% CI** | | ***P*-value** |
|  | 0 | -1.27 | 0.28 | (0.09, | 0.83) | .02 |
|  | 2 | -0.93 | 0.39 | (0.17, | 0.91) | .03 |
|  | 4 | -0.60 | 0.55 | (0.29, | 1.06) | .07 |
|  | 7 | -0.09 | 0.91 | (0.48, | 1.71) | .77 |
|  | 10 | 0.41 | 1.51 | (0.61, | 3.73) | .38 |
|  |  |  |  |  |  |  |
| **Within Group Comparison, describing a one-unit increase in Beck Hopelessness** | **Beck Hopelessness** | **Beta** | **HR** | **95% CI** | | ***P*-value** |
| CATCH-IT | 1 unit change | 0.11 | 1.12 | (0.98, | 1.28) | .10 |
| HE | 1 unit change | -0.06 | 0.95 | (0.85, | 1.06) | .32 |
| Note: Higher CES-D scores indicate higher levels of depression | | | |  |  |  |
| Note: n=7 subjects missing baseline CES-D | |  |  |  |  |  |
| Note: n=99 subjects missing Beck Hopelessness scale | | |  |  |  |  |

**Table S9. Adjusted Cox Proportional Hazard Model for Occurrence of First Depressed Episode (DSR ≥ 3+), Including CRPBI father monitoring subscale as a Moderator, Full Sample (N=369) at 24 months**

| **Model Estimates** |  | **Beta** | **HR** | **95% CI** | | ***P*-value** |
| --- | --- | --- | --- | --- | --- | --- |
| CATCH-IT vs. HE |  | 2.50 | 12.15 | (0.79, | 187.43) | .07 |
| Male vs. Female |  | -1.44 | 0.24 | (0.08, | 0.70) | .009 |
| Non-white vs. white |  | 0.49 | 1.64 | (0.70, | 3.83) | .26 |
| Hispanic vs. not |  | 0.27 | 1.31 | (0.51, | 3.41) | .57 |
| Age at baseline |  | -0.05 | 0.95 | (0.73, | 1.24) | .72 |
| Site (Boston vs. Chicago) |  | 0.08 | 1.08 | (0.50, | 2.36) | .84 |
| Baseline CES-D |  | 0.06 | 1.06 | (0.98, | 1.15) | .14 |
| CPRBI father monitoring |  | 0.07 | 1.07 | (0.90, | 1.27) | .44 |
| CATCH-IT * CPRBI father monitoring Interaction |  | -0.26 | 0.77 | (0.59, | 1.00) | .048 |
| **Between Group Comparisons, comparing CATCH-IT to HE, for increasing CPRBI father monitoring** | **CPRBI father monitoring** | **Beta** | **HR** | **95% CI** | | ***P*-value** |
|  | 5 | 1.19 | 3.29 | (0.71, | 15.14) | .13 |
|  | 10 | -0.12 | 0.89 | (0.43, | 1.85) | .75 |
|  | 12 | -0.64 | 0.53 | (0.22, | 1.26) | .15 |
|  | 15 | -1.42 | 0.24 | (0.06, | 1.02) | .05 |
|  |  |  |  |  |  |  |
| **Within Group Comparison, describing a one-unit increase in CPRBI father monitoring** | **CPRBI father monitoring** | **Beta** | **HR** | **95% CI** | | ***P*-value** |
| CATCH-IT | 1 unit change | -0.19 | 0.82 | (0.68, | 0.99) | .04 |
| HE | 1 unit change | 0.07 | 1.07 | (0.90, | 1.27) | .44 |
| Note: Higher CES-D scores indicate higher levels of depression | | | | | | |
| Note: n=7 subjects missing baseline CES-D | | | | | | |
| Note: n=199 subjects missing CPRBI father monitoring subscale | | | | | | |

**Table S10. Study Variable Construction**

|  | **Measure** | **Variable Construction** |
| --- | --- | --- |
| **Eligibility Variables** |  |  |
| Sub-threshold depressive symptoms | CES-D_10_ or CES-D_20_ | Sub-threshold depressive symptoms were defined as a CES-D_10_ score of 8-17 or CES-D_20_ score of 16 or greater. |
| Prior depressive episode | K-SADS | Prior episodes of depression were determined based on the depression module of the K-SADS. Prior depressive symptoms were rated using the Depression Symptom Rating (DSR). The K-SADS assessment was also used to exclude teens who were in a current depressive episode or who were found to meet criteria for schizophrenia, psychosis, bipolar disorder, or imminent risk of suicide. |
| **Outcomes** |  |  |
| First Depressive Episode | Depression Symptom Rating (DSR) from the K-LIFE | Participants were assessed using the K-LIFE to track the potential onset (or offset) of a depressive episode for each week between assessment timepoints. For each week the assessor would assign a DSR score based on the level and severity of depressive symptoms. A score of 3 or greater indicating the presence of either a sub-threshold or full depressive episode. The first depressive episode was defined as the first time a participant had a DSR score of 3 or higher after the start of the study. |
| Depressive Symptoms | CES-D_10_ | The CES-D_10_ score was used to track depressive symptoms at baseline, two months, six months, 12 months, and 24 months. |
| Mental Health Functioning | GAS | GAS scores were recorded at each assessment to measure global mental health functioning. |
| **Moderators** |  |  |
| Participant Characteristics | Demographics | Moderator analyses included sex (female/male), race (white/not white), ethnicity (Hispanic/Non-Hispanic), age (continuous variable), maternal education (college/no college), and site (Chicago/Boston). |
| Vulnerability Factors | CES-D_10_ | The total score for the CES-D_10_ was used for the moderator analysis. |
|  | Adolescent Life Events Questionnaire (ALEQ) | The total score for the ALEQ was used for the moderator analysis. |
|  | **Measure** | **Variable Construction** |
|  | Beck Hopelessness Scale (BHS) | The total score for the BHS was used for the moderator analysis. |
|  | Child Report of Parental Behavior Inventory (CRPBI) | The subscale scores for maternal acceptance, maternal control, maternal monitoring, paternal acceptance, paternal control, paternal monitoring of the CRPBI were used for the moderator analysis. |
| Motivation | Theory of Planned Behavior Scale | The total score for the Theory of Planned Behavior Scale was used for the moderator analysis. |
|  | Trans-theoretical Model Scale | The total score for the Trans-theoretical Model Scale was used for the moderator analysis. |
| Physician Relationship | Physician Relationship Scale | The total score for the Physician Relationship Scale was used for the moderator analysis. |
| Teen Anxiety | Screen for Child Anxiety Related Emotional Disorders (SCARED) | The total score for the SCARED was used for the moderator analysis. |
| Teen Behavior Problems | Disruptive Behaviors Disorder Scale (DBD) | The AHDH subscale and OD/CD subscale scores of the DBD were used for the moderator analysis. |
| Teen Substance Abuse | CRAFFT | The total score for the CRAFFT was used for the moderator analysis. |
| Parent Depressive Symptoms | CES-D_10_ | The CES-D_10_ was also administered to a parent of each participant. The total score was used in the moderator analysis. |
| Participant Adherence | Program metrics | Adherence was broken down into three variables. 1) Modules completed by the teen, 2) Modules completed by the parent, 3) Modules completed by both the teen and the parent. |

**Table S11. Motivational Interviews, CATCH-IT Participants**

| **Motivational Interviews** | **Mean or N** | **SD  or %** |
| --- | --- | --- |
| **Adolescent MIs completed (N=193)** |  |  |
| Mean (SD) | 2.0 | (0.9) |
| Number completed, N (%) |  |  |
| 0 | 10 | (5.2%) |
| 1 | 53 | (27.5%) |
| 2 | 64 | (33.2%) |
| 3 | 66 | (34.2%) |
| **Parent MIs completed (N=165)** |  |  |
| Mean (SD) | 1.9 | (0.9) |
| Number completed, N (%) |  |  |
| 0 | 10 | (6.1%) |
| 1 | 50 | (30.3%) |
| 2 | 54 | (32.7%) |
| 3 | 51 | (30.9%) |
| **MI Fidelity: Mean (SD) MITI Summary Scores (N=32)** |  |  |
| Technical Global (1-5) | 3.0 | (0.5) |
| Relational Global (1-5) | 15.7 | (8.0) |
| %CR | 25.7 | (24.2) |
| R:Q | 0.5 | (0.3) |
| Total MI-Adherent | 0.4 | (0.7) |
| Total MI-Non-Adherent | 0.9 | (1.3) |
| **MI duration, minutes (N=28)** |  |  |
| Mean (SD) | 7.7 | (4.0) |
| Median | 6.9 |  |

Definitions:

Technical Global: (cultivating change talk+softening sustain talk)/2, 1-5, higher is better

Relational Global: (partnership+empathy)/2, 1-5, higher is better

% CR: 100*complex reflection/(simple reflection+complex reflection)

Reflection to Question Ratio: total reflections (simple+complex) / total questions

Total MI-adherent: seeking collaboration+affirm+emphasizing autonomy

Total MI non-adherent: confront+persuade

**Table S12. Participant Contacts: Telephone Calls and Emails, Chicago Participants**

|  | **All (N=248)** | | **CATCH-IT (N=131)** | | **Health Education (N=117)** | |  |
| --- | --- | --- | --- | --- | --- | --- | --- |
| **Contacts** | **Mean** | **SD** | **Mean** | **SD** | **Mean** | **SD** | ***P*^b^** |
| Contacts and attempts^a^ | 32.3 | (14.8) | 36.1 | (15.7) | 28.1 | (12.6) | <.001 |
| Contacts | 15.7 | (8.0) | 17.0 | (8.9) | 14.1 | (6.5) | .004 |
| Contacts by time from baseline |  |  |  |  |  |  |  |
| 0-6 months | 7.4 | (4.2) | 8.6 | (4.7) | 6.0 | (3.1) | <.001 |
| >6-12 months | 3.0 | (2.1) | 2.9 | (2.2) | 3.1 | (2.0) | .53 |
| >12 months | 5.3 | (4.2) | 5.5 | (4.6) | 5.0 | (3.8) | .37 |
| ^a^ From contact log maintained by Chicago staff. The log included administrative contacts such as scheduling calls in addition to coaching calls, safety checks, and motivational interview calls. Contacts were defined as cases where staff spoke with the participant or received email from the participant. Attempts were defined as calls that did not reach the participant directly (though a message was usually left if possible) or email messages with no response.  ^b^ From t-tests with pooled variance. | | | | | | | |

**Table S13. Total Time: Assessment and Follow-up, Chicago Participants**

|  | **All**  **(N=40)** | |
| --- | --- | --- |
| **K-SADS duration, minutes** | **Mean** | **SD** |
| Mean (SD) |  |  |
| Baseline | 38.9 | (15.8) |
| 2 months | 15.4 | (13.0) |
| 6 months | 12.6 | (10.0) |
| 12 month | 14.5 | (10.9) |
| 24 months | 20.0 | (11.5) |

^a^N=40 for estimated assessment and follow-up duration

**Table S14. Current or Past Episodes at Baseline, by Group**

|  | **CATCH-IT (N=190)** | | **Health Education (N=175)** | |  |
| --- | --- | --- | --- | --- | --- |
|  | **N** | **%** | **N** | **%** | ***P*^a^** |
| **Current or past episode** |  |  |  |  | .56 |
| DSR ≥ 4 | 76 | (40.0%) | 71 | (40.6%) |  |
| DSR 3 | 41 | (21.6%) | 45 | (25.7%) |  |
| No episode | 73 | (38.4%) | 59 | (33.7%) |  |
| ^a^ From Cochran-Mantel-Haenszel test for difference in row mean scores. Four cases with missing data for current and past DSR at baseline are excluded from the analysis. | | | | | |

**Table S15. Time to First Episode (DSR ≥3) Among Adolescents with Past or Current DSR ≥3 at Baseline**

| **Time to first episode** | **CATCH-IT (N=117)** | | **Health Education (N=116)** | |
| --- | --- | --- | --- | --- |
|  | N | % | N | % |
| ≤ 2 mon | 4 | (3.4%) | 4 | (3.4%) |
| > 2 mon | 17 | (14.5%) | 19 | (16.4%) |
| No episode | 96 | (82.1%) | 93 | (80.2%) |

**Table S16. Adolescents with Episodes (DSR ≥ 3) at Follow-up by Most Severe Current or Past DSR at Baseline**

|  | **CATCH-IT** | | | **Health Education** | | |
| --- | --- | --- | --- | --- | --- | --- |
| **Current or past DSR at baseline** | **Total N** | **N with follow-up episodes** | **% with follow-up episodes** | **Total N** | **N with follow-up episodes** | **% with follow-up episodes** |
| Current/past BL DSR ≥ 4 | 76 | 15 | (19.7%) | 71 | 12 | (16.9%) |
| Current/past BL DSR ≥ 3 | 117 | 21 | (17.9%) | 116 | 23 | (19.8%) |
| No current or past episode | 73 | 6 | (8.2%) | 59 | 10 | (16.9%) |

**Table S17. Functional status scores from baseline to 24 months**

|  | **Unadjusted means** | | | | | |  | **Within-group slopes^a^** | | | | | | **Between- grp diff in slopes^b^** |
| --- | --- | --- | --- | --- | --- | --- | --- | --- | --- | --- | --- | --- | --- | --- |
|  | **CATCH-IT (N=193)** | | | **Health Education (N=176)** | | |  | **CATCH-IT** | | | **Health Education** | | |  |
|  | **N** | **Mean** | **SD** | **N** | **Mean** | **SD** | **Time variable^c^** | **b** | **SE** | ***P*** | **b** | **SE** | ***P*** | ***P*** |
| **GAS (1-100)** |  |  |  |  |  |  | Months | 0.398 | (0.041) | <.001 | 0.400 | (0.039) | <.001 | .97 |
| Baseline | 193 | 78.3 | (9.3) | 174 | 78.0 | (9.6) | Log(months+1) | 2.964 | (0.285) | <.001 | 3.076 | (0.278) | <.001 | .78 |
| 2 months | 114 | 83.4 | (8.6) | 135 | 82.8 | (11.5) |  |  |  |  |  |  |  |  |
| 6 months | 126 | 84.6 | (8.9) | 136 | 84.5 | (10.2) |  |  |  |  |  |  |  |  |
| 12 months | 120 | 86.1 | (9.3) | 139 | 86.2 | (10.2) |  |  |  |  |  |  |  |  |
| 24 months | 82 | 87.8 | (8.8) | 98 | 88.3 | (9.1) |  |  |  |  |  |  |  |  |
| ^a^ From linear mixed effect growth models with random intercept and slope, adjusted for sex, ethnicity (Hispanic, non-Hispanic), race (white, non-white), baseline age, site, and baseline teen CES-D_10_. Within-group estimated slopes and p-values are from estimates of simple slopes.  ^b^ The p-value for the group*time interaction is used to test for a significant difference between slopes.  ^c^ Models were run with and without transformation of the time variable to improve linearity. | | | | | | | | | | | | | | |

**Table S18. Summary of Growth Curve Moderator Analyses: P-values for Interaction Term, with and without Time Transformation**

| **Moderator** | **Participants included in analyses** | **No transformation p for group*time*moderator** | **Transformation of time term p for group*time*moderator** |
| --- | --- | --- | --- |
| Parent+teen modules completed | 362 | .37 | .89 |
| Teen modules completed | 362 | .46 | .95 |
| Parent modules completed | 340 | .73 | .96 |
| Parent+teen modules completed, square-root transformed | 362 | (Not run) | .92 |
| Teen modules completed, square-root transformed | 362 | (Not run) | .64 |
| Parent modules completed, square-root transformed | 340 | (Not run) | .92 |
| Parent CES-D10, baseline | 332 | .06 | .09 |
| Teen GAS, baseline | -- | -- | -- |
| Teen CES-D10, baseline | 362 | .49 | .65 |
| Sex | 362 | .84 | .96 |
| Race | 362 | .09 | .19 |
| Ethnicity | 362 | .18 | .28 |
| Site | 362 | .91 | .76 |
| Maternal education | 352 | .53 | .50 |
| Theory of planned behavior, bl | 164 | .96 | .93 |
| Stressful life events (LEQ), bl | 300 | .10 | .23 |
| Hopelessness (BHS), baseline | 270 | .93 | .78 |
| Trans-theoretical model, bl | 191 | .41 | .43 |
| Positive relationships in primary care, 2 months | 133 | .83 | .83 |
| Anxiety (SCARED), baseline | 311 | .63 | .52 |
| CRAFFT, baseline | 278 | .29 | .23 |
| ADHD (DBD-A), baseline | 196 | .42 | .08 |
| ODCD (DBD-A), baseline | 193 | .63 | .88 |
| Social adjustment (SAS-SR), bl | 212 | .53 | .53 |
| Maternal acceptance (CRPBI), bl | 196 | .30 | .44 |
| Maternal control (CRPBI), bl | 197 | .47 | .70 |
| Maternal monitoring (CRPBI), bl | 187 | .03 | .16 |
| Paternal acceptance (CRPBI), bl | 176 | .78 | .60 |
| Paternal control (CRPBI), bl | 176 | .79 | .85 |
| Paternal monitoring (CRPBI), bl | 170 | .36 | .39 |

**Table S19. Participant Demographics at Baseline**

|  | **All (N=369)** | | | **Chicago (N=248)** | | | **Boston (N=121)** | | |  |
| --- | --- | --- | --- | --- | --- | --- | --- | --- | --- | --- |
|  | **N** | **Mean or %** | **(SD or N)** | **N** | **Mean or %** | **(SD or N)** | **N** | **Mean or %** | **(SD or N)** | **p^a^** |
| Teens | 369 |  |  | 248 |  |  | 121 |  |  |  |
| Age, years | 369 | 15.4 | (1.5) | 248 | 15.5 | (1.5) | 121 | 15.3 | (1.5) | .34 |
| Sex | 369 |  |  | 248 |  |  | 121 |  |  | .13 |
| Female |  | 68.0% | (251) |  | 70.6% | (175) |  | 62.8% | (76) |  |
| Male |  | 32.0% | (118) |  | 29.4% | (73) |  | 37.2% | (45) |  |
| Ethnicity | 369 |  |  | 248 |  |  | 121 |  |  | .01 |
| Hispanic |  | 20.9% | (77) |  | 24.6% | (61) |  | 13.2% | (16) |  |
| Not Hispanic^b^ |  | 79.1% | (292) |  | 75.4% | (187) |  | 86.8% | (105) |  |
| Race | 369 |  |  | 248 |  |  | 121 |  |  | <.0001 |
| White |  | 54.5% | (201) |  | 46.8% | (116) |  | 70.2% | (85) |  |
| Not white^c^ |  | 45.5% | (168) |  | 53.2% | (132) |  | 29.8% | (36) |  |
| Mother's education | 359 |  |  | 242 |  |  | 117 |  |  | .03 |
| Some HS |  | 3.3% | (12) |  | 4.1% | (10) |  | 1.7% | (2) |  |
| HS graduate/GED |  | 12.5% | (45) |  | 13.2% | (32) |  | 11.1% | (13) |  |
| Some college |  | 24.2% | (87) |  | 27.3% | (66) |  | 17.9% | (21) |  |
| College graduate |  | 59.9% | (215) |  | 55.4% | (134) |  | 69.2% | (81) |  |
| Father's education | 336 |  |  | 227 |  |  | 109 |  |  | <.0001 |
| Some HS |  | 7.7% | (26) |  | 10.6% | (24) |  | 1.8% | (2) |  |
| HS graduate/GED |  | 22.6% | (76) |  | 26.0% | (59) |  | 15.6% | (17) |  |
| Some college |  | 16.4% | (55) |  | 19.8% | (45) |  | 9.2% | (10) |  |
| College graduate |  | 53.3% | (179) |  | 43.6% | (99) |  | 73.4% | (80) |  |
| Marital status of parents | 363 |  |  | 246 |  |  | 117 |  |  | .03 |
| Married |  | 60.6% | (220) |  | 55.7% | (137) |  | 70.9% | (83) |  |
| Divorced |  | 12.7% | (46) |  | 13.8% | (34) |  | 10.3% | (12) |  |
| Separated |  | 6.1% | (22) |  | 5.7% | (14) |  | 6.8% | (8) |  |
| Widowed |  | 3.0% | (11) |  | 3.3% | (8) |  | 2.6% | (3) |  |
| Never married |  | 17.6% | (64) |  | 21.5% | (53) |  | 9.4% | (11) |  |
| Number of siblings | 364 |  |  | 246 |  |  | 118 |  |  | <.0001 |
| 0 |  | 7.7% | (28) |  | 4.5% | (11) |  | 14.4% | (17) |  |
| 1 |  | 34.3% | (125) |  | 28.5% | (70) |  | 46.6% | (55) |  |
| 2 |  | 23.9% | (87) |  | 23.2% | (57) |  | 25.4% | (30) |  |
| ≥3 |  | 34.1% | (124) |  | 43.9% | (108) |  | 13.6% | (16) |  |
| Firstborn child | 367 | 48.2% | (177) | 246 | 45.9% | (113) | 121 | 52.9% | (64) | .21 |
| Times moved or changed houses in lifetime, median and quartile range | 353 | 2.0 | (3.0) | 239 | 2.0 | (3.0) | 114 | 1.0 | (3.0) | .049 |
| Parents | 322 |  |  | 211 |  |  | 111 |  |  |  |
| Age at baseline | 300 | 46.7 | (7.2) | 191 | 45.7 | (6.9) | 109 | 48.6 | (7.5) | .0005 |
| Sex | 322 |  |  | 211 |  |  | 111 |  |  | .63 |
| Female |  | 89.4% | (288) |  | 90.0% | (190) |  | 88.3% | (98) |  |
| Male |  | 10.6% | (34) |  | 10.0% | (21) |  | 11.7% | (13) |  |
| ^a^ From chi-square tests for categorical variables, Cochran-Mantel-Haenszel tests of row mean scores for education variables, Wilcoxon tests for number of siblings and times moved, and t-tests with pooled variance for age.  ^b^ Participants with missing ethnicity data were coded as not Hispanic (N=6).  ^c^ Participants with missing race data were coded as not white (N=20; most identified as Hispanic). | | | | | | | | | | |
